# Gastric cancer causing Schnitzler’s metastasis: case report and systematic review of the features

**DOI:** 10.2340/1651-226X.2025.41296

**Published:** 2025-02-26

**Authors:** Huimin Xue, Xiaomei Yang, Qing Shen, Jinglei Qu, Xiujuan Qu, Ying Chen

**Affiliations:** a Department of Medical Oncology, The First Hospital of China Medical University, Shenyang, People’s Republic of China; b Key Laboratory of Anticancer Drugs and Biotherapy of Liaoning Province, The First Hospital of China Medical University, Shenyang, People’s Republic of China; c Liaoning Province Clinical Research Center for Cancer, Shenyang, People’s Republic of China; d Key Laboratory of Precision Diagnosis and Treatment of Gastrointestinal Tumors, Ministry of Education, The First Hospital of China Medical University, Shenyang, People’s Republic of China

**Keywords:** Schnitzler’s metastasis, rectal metastasis from gastric cancer, clinicopathological characteristics, case report, systematic review

## Abstract

**Background:**

Rectal metastasis from gastric cancer (GC), also known as Schnitzler’s metastasis, is a rare phenomenon. The clinicopathological characteristics, outcomes, and prognostic factors of this condition remain poorly understood.

**Methods:**

We describe a case of GC causing Schnitzler’s metastasis and present a systematic review on case reports and case series. Data extracted and analyzed include clinicopathological features, treatment modalities received, outcomes, and follow-up.

**Results:**

A total of 34 records, including our own, encompassing 41 cases were incorporated into the study. The median age of patients at admission was 59 years, with females accounting for 53.7% of cases. The predominant histological subtype of Schnitzler’s metastasis was moderate-to-poorly differentiated adenocarcinoma, representing 31 cases (86.1%). Among the patients in this cohort, 38.9% exhibited signet-ring cell carcinoma. Regarding the initial diagnosis of GC, 28.6% were categorized as stage IIIA, and 28.6% were classified as stage IV. The median overall survival (OS) time was 72 months (95% confidence interval [CI]: 27-NA), while the median OS since the diagnosis of metastatic cancer was 16 months (95% CI: 9-NA).

**Interpretation:**

Schnitzler’s metastasis presents a challenge in the pathology of colorectal endoscopy and may lead to treatment delays. Imaging features such as increased thickness of the intestinal wall and significant layered enhancement can aid in diagnosis; however, deep core biopsy of intestinal lesions remains the gold standard for diagnosing rectal metastases. Accurately distinguishing rectal metastases from primary rectal cancer is crucial for preventing unnecessary therapeutic interventions.

## Introduction

Globally, gastric cancer (GC) is the fifth most common cancer and the fourth leading cause of cancer death, accounting for 7.7% of cancer fatalities [1]. After radical resection of GC, it commonly spreads through lymphatic, peritoneal, hematogenous routes, or local recurrence, often affecting the liver, lung, bone, lymph nodes, and brain. Rectal metastasis, termed Schnitzler’s metastasis, is exceedingly rare. Given the unique site of metastasis, distinguishing between GC intestinal metastasis and primary colorectal cancer poses a clinical challenge.

Rectal metastasis presents symptoms similar to primary rectal cancer, such as abdominal pain, ascites, constipation, diarrhea, and severe cases of bowel obstruction and bleeding, leading to reduced quality of life and poor treatment outcomes [2]. Diagnosis primarily involves digital rectal examination, colonoscopy, and endoscopic ultrasonography (EUS), with computed tomography (CT) and fluorodeoxyglucose positron emission tomography/computed tomography (FDG PET/CT) scans essential for identifying metastases. Biopsies can be challenging, often requiring deep tissue samples to confirm malignancy.

This article reviews global case reports and series on Schnitzler’s metastasis, focusing on its clinical presentation, imaging, histopathology, treatment, and prognosis. It aims to improve clinical understanding and management of rectal metastases from GC by consolidating existing knowledge.

## Methods

We present a case of rectal metastasis from GC treated at our institution and conducted a systematic review of case reports and series on GC causing Schnitzler’s metastasis. The search strategies apply the following terms: ‘Stomach Neoplasms’ (Mesh term), ‘Rectum’ (Mesh term), ‘Neoplasm Metastasis’ (Mesh term), ‘Stomach Carcinoma’ (free text), ‘Stomach Cancer’ (free text), ‘Stomach Neoplasm’ (free text), ‘rectum’ (free text), and ‘Metastasis’ (free text). These terms were combined using ‘AND’ and ‘OR’. The databases ‘PubMed’ (https://pubmed.ncbi.nlm.nih.gov/), ‘Embase’ (https://www.embase.com/), ‘CNKI’ (https://www.cnki.net/), and ‘Cochrane’ (https://www.cochrane.org/) were thoroughly reviewed.

On November 13, 2023, investigators HX and XY independently reviewed relevant articles, resolving discrepancies through discussion. Studies were excluded if off-topic, duplicates, lacking full text, or insufficient clinical data, and references were checked to avoid omissions. Figure 3 illustrates the selection process per PRISMA guidelines [3]. As all articles were case reports, no risk bias assessment tool was used. Extracted data included authorship, publication year, patient demographics, clinical details, and outcomes, which were then synthesized and analyzed.

## Statistical analysis

Descriptive data were presented as median (interquartile range) or number and percentages. The overall survival (OS) was measured from the date of primary GC diagnosis to the date of death, while survival subsequent to rectal metastasis was calculated from the date of metastasis diagnosis to the date of death. Survival data were analyzed using the Kaplan–Meier method. Statistical analyses were performed using R software (Version 4.3.3; The R Foundation for Statistical Computing, Vienna, Austria).

## Results

### Case report

In December 2019, a 39-year-old woman was diagnosed with stage IIA GC (T3N0M0) and human epidermal growth factor receptor 2 (HER-2) negative. She had a total gastrectomy, six cycles of S-1 and oxaliplatin (SOX) chemotherapy, and one cycle of tegafur gimeracil and oteracil potassium capsules (S-1) monotherapy. By June 2021, she reported defecation difficulties, decreased stool caliber, and increased bowel movement frequency.

**Figure 1:**
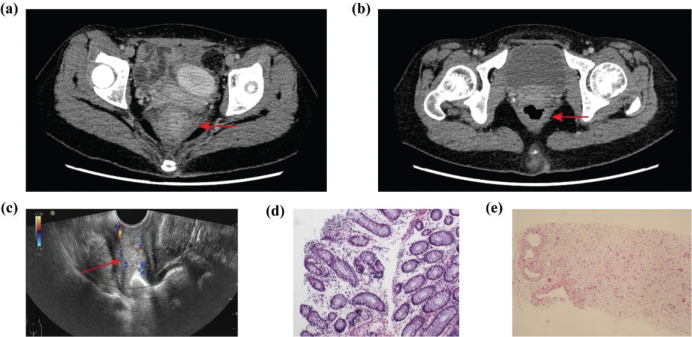
(a) Enhanced abdominal CT scan on Jun. 2021 showed thickening and circumferential enhancement of the rectal wall (red arrow). (b) Enhanced abdominal CT scan after treatment on Dec. 2021. (c) Endorectal EUS showed a thickened rectal wall, narrowing of the intestinal lumen, and increased blood flow in the thickened wall (red arrow). (d) Superficial biopsy from colonoscopy revealed nonspecific chronic inflammatory changes. (e) Deep puncture biopsy from endorectal EUS showed poorly differentiated adenocarcinoma originating from stomach (hematoxylin and eosin stain). CT, Computed Tomography; EUS, Endoscopic Ultrasound.

**Figure 2:**
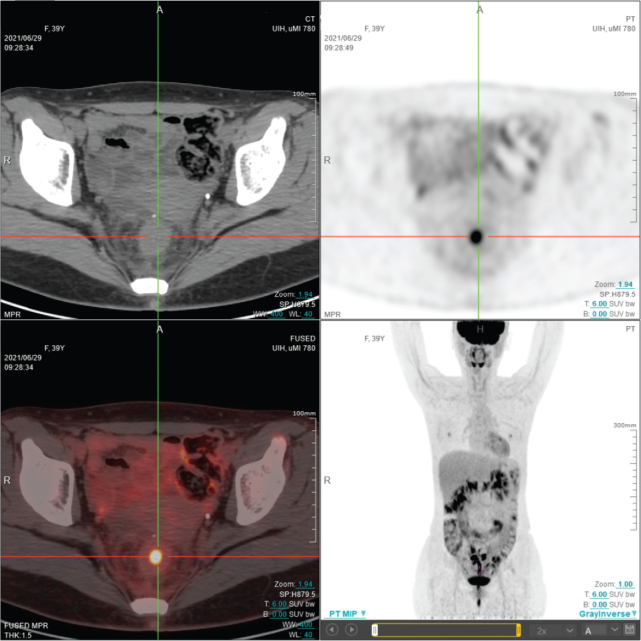
FDG-PET showed hypermetabolism in the middle section of rectum on Jun. 2021. FDG: F-18-fluoro-deoxy-glucose; PET: positron emission tomography.

A follow-up enhanced abdominal CT scan revealed rectal wall thickening and circumferential enhancement (Figure 1a). An FDG PET/CT scan showed increased metabolic activity in the rectum, with a standardized uptake value (SUV) of 11.5 (Figure 2). A colonoscopy at another hospital found irregular, friable mucosa prone to bleeding from 4 to 10 cm from the anal verge. A superficial biopsy indicated nonspecific chronic inflammation (Figure 1d). Intraluminal ultrasonography confirmed a thickened rectal wall, narrowed lumen, and increased blood flow (Figure 1c). An ultrasonography-guided core needle biopsy using an 18G coaxial cutting needle diagnosed poorly differentiated adenocarcinoma from the stomach (Figure 1e). So, the patient was diagnosed with Schnitzler’s metastasis.

**Figure 3:**
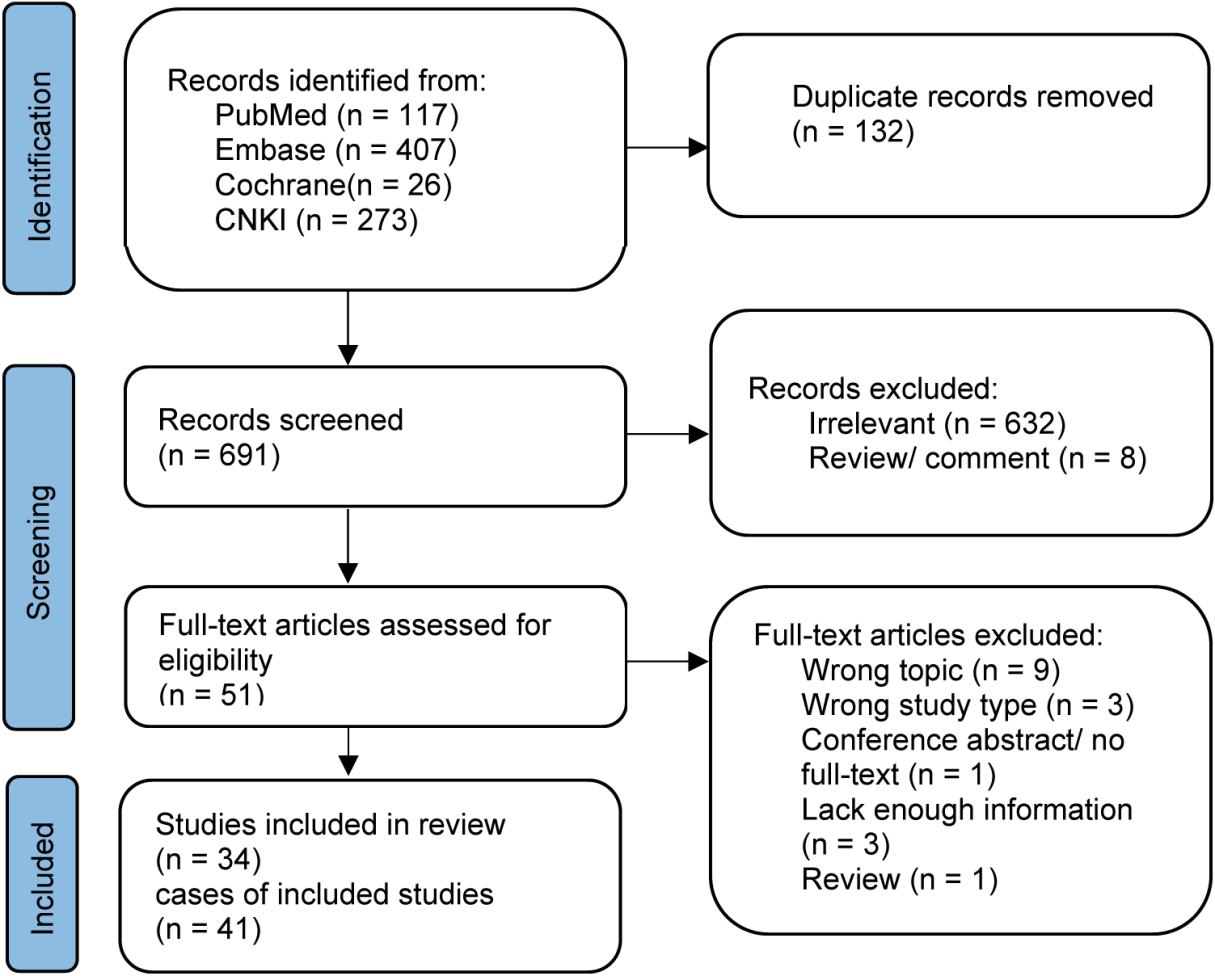
Flowchart of the selection process according to PRISMA statement.

Immunohistochemistry showed C-erbB-2(2+), while fluorescence in situ hybridization (FISH)-HER-2 was negative. The patient received chemotherapy with apatinib and albumin-bound paclitaxel from August 2021 to June 2022, maintaining stable disease. In July 2022, disease progression occurred, prompting 27 cycles of disitamab vedotin for rectal metastasis with HER-2 2+ status. Apatinib was added in December 2022 for combined therapy. As of October 2023, no tumor recurrence was detected. This study complies with CARE guidelines, and the patient consented to the case report [4].

### Literature review

**Figure 4:**
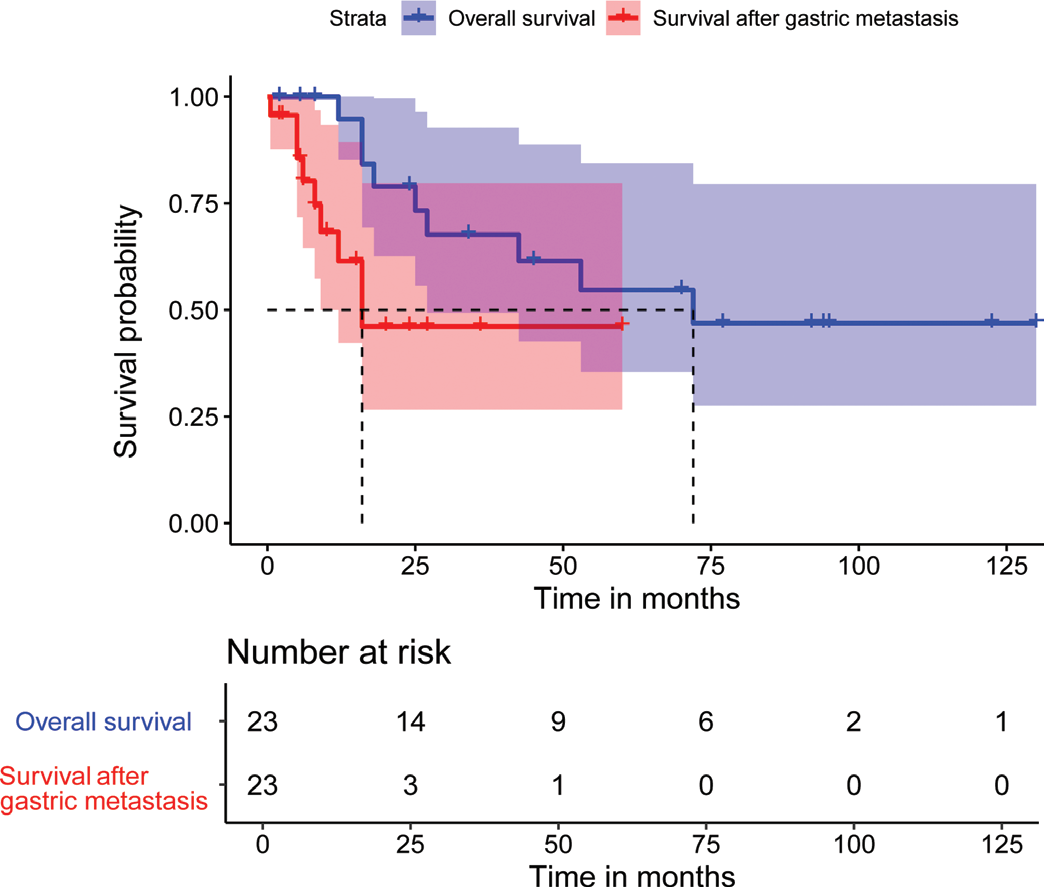
Kaplan–Meier curves for overall survival time and survival time after GC Schnitzler’s metastasis. The median OS of patients was 72 months (95% CI: 27-NA), and the survival time after the diagnosis of Schnitzler’s metastatic cancer was 16 months (95% CI: 9-NA). GC, gastric cancer.

A total of 41 cases were included in this review, combining 40 from the literature with our case (Figure 3) [5–37]. The median participant age was 59 years, with 46.3% male and 53.7% female. Geographically, cases came from Japan (36.6%), China (31.7%), the USA (17.1%), Turkey (7.3%), and one each from Germany, Italy, and South Korea. Most cases involved moderate-to-poorly differentiated adenocarcinoma (86.1%), with 38.9% being signet-ring cell carcinoma and 36.1% poorly differentiated adenocarcinoma. Highly differentiated adenocarcinoma appeared in only 2.8% of cases. Initial GC diagnoses were stage IIIA or IV in 28.6% of cases each. Among the 29 cases that specified the number of metastatic sites, nine cases (31.0%) presented with metastasis at a single site at the time of diagnosis, while 20 cases (69.0%) reported metastases other than rectum metastases. Synchronous metastases accounted for 16 cases (40.0%), while metachronous metastases were observed in 24 cases (60.0%) (Table 1). The predominant symptoms associated with Schnitzler’s metastasis included mechanical bowel obstruction, manifested as abdominal mass, distension, discomfort, and/or pain (57.5%), followed by constipation (52.5%), rectal discharge or hematochezia (17.5%), and nausea, dyspepsia, or vomiting (10.0%). The median distance of the rectal mass from the anal verge was 4 cm. Diagnostic modalities included endoscopy in 80.5% of cases, EUS in 7.3%, CT scan in 70.7%, and magnetic resonance imaging in 22.0%. PET/CT scan was utilized in 17.1% of cases (Table 2). For primary stomach cancer, the most common treatment was surgery alone (61.1%), followed by chemotherapy (25.0%), surgery with chemotherapy (11.1%), and surgery with chemoradiation (2.8%). The treatment modalities for rectal metastasis include surgery alone (46.9%), chemotherapy (25.0%), a combination of surgery and chemotherapy (25.0%), and a combination of chemotherapy and targeted therapy (2.8%). As for outcomes, 60.9% of patients were alive, while 39.1% succumbed to the disease with a median follow-up of 9 months (interquartile range, 5–20) after Schnitzler’s metastasis. The median OS of patients was 72 months (95% confidence interval [CI]: 27-NA), and the survival time after the diagnosis of metastatic cancer was 16 months (95% CI: 9-NA) (Figure 4, Table 3).

**Table 1:** Demographic and clinicopathologic features of GC Schnitzler’s metastasis.

Characteristics	Value
**Age (years)**	59 (53–68.5)
**Gender**	
Men	19 (46.3)
Woman	22 (53.7)
**Country**	
China	13 (31.7)
Germany	1 (2.4)
Italy	1 (2.4)
Japan	15 (36.6)
Korea	1 (2.4)
Turkey	3 (7.4)
United States	7 (17.1)
**Histological type (36 cases available)**	
Moderate-to-poorly differentiated adenocarcinoma	31 (86.1)
SRCC	14 (38.9)
Poorly differentiated adenocarcinoma	13 (36.1)
Highly differentiated adenocarcinoma	1 (2.8)
Others	4 (11.1)
**T stage (20 cases available)**	
T1	2 (10.0)
T2	2 (10.0)
T3	8 (40.0)
T4	8 (40.0)
**N stage (20 cases available)**	
N0	10 (50.0)
N+	10 (50.0)
**M stage (20 cases available)**	
M0	14 (70.0)
M+	6 (30.0)
**AJCC stage (21 cases available)**	
IA	2 (9.5)
IB	2 (9.5)
IIA	2 (9.5)
IIB	1 (4.8)
IIIA	6 (28.6)
IIIB	2 (9.5)
IIIC	1 (4.8)
IV	5 (23.8)
**No. of metastasis sites (29 cases available)**	
Solitary	9 (31.0)
Multiple	20 (69.0)
**Interval (40 cases available)**	
Synchronous	16 (40.0)
Metachronous	24 (60.0)

Data presented as the number of patients (%) or median (interquartile range).

GC: gastric cancer; SRCC: Signet ring cell carcinoma; AJCC: American Joint Committee on Cancer.

**Table 2:** Clinical presentation and diagnosis workup of GC Schnitzler’s metastasis tumors.

Characteristics	Value
**Clinical presentation (40 cases available)**	
Abdominal distention and/or pain	23 (57.5)
Constipation or difficulty defecating	21 (52.5)
Ascites	1 (2.5)
Dyspepsia, nausea, or vomiting	4 (10.0)
Weight loss	3 (7.5)
Rectal tenesmus	3 (7.5)
Rectal discharge or hematochezia	7 (17.5)
Anemia	2 (5.0)
Little stool output	3 (7.5)
Rectal mass	1 (2.5)
Diarrhea	3 (7.5)
Fatigue	1 (2.5)
Asymptomatic	1 (2.5)
**Distance from the anal verge (17 cases available)**	4 (4–6.5)
**Diagnosis workup (41 cases available)**	
Digital rectal examination	16 (39.0)
Endoscopy	33 (80.5)
EUS	3 (7.3)
CT	29 (70.7)
PET scan	7 (17.1)
MRI	9 (22.0)

Data presented as the number of patients (%) or median (interquartile range).

GC: gastric cancer; EUS: Endoscopic Ultrasound; CT: Computed Tomography; PET: Positron Emission Tomography; MRI: Magnetic Resonance Imaging.

**Table 3:** Treatment and prognosis features of primary and GC Schnitzler’s metastasis tumors.

Characteristics	Value
**Primary stomach treatment (36 cases available)**	
Surgery alone	22 (61.1)
Chemotherapy alone	9 (25.0)
Chemotherapy + targeted therapy	1 (2.8)
Surgery + chemotherapy	4 (11.1)
Surgery + chemoradiation	1 (2.8)
**Rectal metastasis treatment (32 cases available)**	
Surgery alone	15 (46.9)
Chemotherapy alone	8 (25.0)
Chemotherapy + targeted therapy	3 (9.4)
Chemoradiation	1 (3.1)
Surgery + chemotherapy	8 (25.0)
Surgery + chemoradiation	1 (3.1)
**Survival information (23 cases available)**	
Dead	9 (39.1)
Alive	14 (60.9)
Survival after diagnosis of primary cancer, months	72 (27-NA)
Survival after diagnosis of metastatic cancer, months	16 (9-NA)

Data presented as the number of patients (%) or median (interquartile range).

GC: gastric cancer.

## Discussion

Rectal metastasis from GC, also known as Schnitzler’s metastasis, is underrepresented in medical literature, leading to potential oversight in clinical practice. Giri et al. reported a case of pancreatic carcinoma causing Schnitzler’s metastasis [38], expanding the definition of Schnitzler’s metastasis. Considering the diagnostic and therapeutic challenges, we described a case of rectum metastasis from gastric adenocarcinoma, which was confirmed by CT, PET-CT, endoscopy, EUS, tissue biopsy, and immunohistochemistry. Additionally, 41 cases were systematically analyzed to elucidate their clinical characteristics.

Schnitzler’s metastasis is primarily reported in Japan and China due to high rates of gastrointestinal tumors in East Asia. It is more common in females, with our study showing 53.7% female patients. Primary GC often involves moderate-to-poorly differentiated adenocarcinoma, with 70% of our cases having poorly differentiated tumor cells and 30% having signet-ring cells. Additionally, 69% of patients had multiple metastases, likely due to advanced primary GC. Rectal metastases can occur at initial diagnosis or during recurrence, with 40% synchronous and 60% heterochronous cases in our study.

The precise mechanism underlying Schnitzler’s metastasis remains unclear. Detecting tumor cells in rectal biopsies is challenging, possibly due to the spread of gastric adenocarcinoma through rectal submucosa veins and lymphatics, and the sparse tumor cell distribution in gastric impression cell carcinoma [5]. Additionally, intestinal wall thickening might result from fibrous tissue and inflammatory cell accumulation due to lymphatic obstruction by cancer cells causing edema, rather than direct tumor cell infiltration [18]. Peritoneal dissemination, where cancer cells accumulate in the uterorectal or vesicorectal crypts leading to implantation metastasis, is another possible explanation [37]. The ‘dormancy’ theory suggests that malignant cells spread early, remain inactive in distant organs, and reactivate under stress, explaining the long latency between initial cancer and distant metastasis.

Distinguishing between recurrent intestinal metastases and primary colorectal cancer post-stomach resection is difficult due to overlapping symptoms like abdominal pain and bowel issues. Tang et al. described a case misdiagnosed as rectal cancer, resulting in unnecessary radiation therapy [32]. Our study found rectal stenosis blocking fecal passage due to metastasis, along with changes in bowel habits.

### Diagnosis

Digital rectal examination is crucial for detecting rectal issues, with palpation revealing severe stenosis 5–6 cm from the dentate line [7]. Colonoscopy, performed in 80.5% of cases, typically shows diffuse wall thickening [39]. The median mass distance from the anal verge is 4 cm. EUS is recommended for a detailed view of rectal layers, detecting tumor infiltration even with normal mucosa [40].

CT imaging typically reveals thickening and circumferential enhancement of the bowel wall, often characterized by homogeneous thickening and stratified enhancement [41]. The abnormal thickening of the bowel wall has been attributed to the infiltration of cancer cells into the mesenchyme and the subsequent formation of fibrous tissue within the bowel wall. However, the limited resolution of CT precludes differentiation of the individual layers of the bowel wall. FDG PET/CT is vital for detecting recurrent metastases after GC surgery and staging advanced cases, often identifying lesions missed by other imaging methods [42]. Lim et al. and Dogan et al. highlighted cases of rectal hypermetabolism with SUVs of 7.6 and 5.8, respectively [7, 10]. In our case, we noted diffuse FDG uptake in the recto-anal area with a maximum SUV of 11.4. The use of FDG PET/CT for Schnitzler’s metastases could provide insights into intestinal obstruction after radical gastrectomy for GC, aiding treatment strategies.

The diagnosis of submucosal lesions through endoscopic mucosal biopsy can be challenging, with some studies confirming diagnoses via surgical pathology [5, 18, 22]. We recommend intraluminal ultrasound-guided deep biopsy as it facilitates the diagnosis of submucosal lesions while minimizing tissue damage. Imaging showed rectal wall thickening, and difficulty in tumor cell identification on biopsy should raise suspicion of Schnitzler metastasis. A detailed patient history and further investigations, including FDG PET/CT and deep biopsies, are essential to determine the lesion’s nature.

### Treatment

The management of Schnitzler’s metastasis should consider factors such as location, extent, histological type, and primary tumor stage. Patients with colonic metastases occurring 5 years after aggressive GC surgery, who underwent surgical intervention, showed an average survival of 26 months [17]. Decompression therapy, such as colostomy or colonic stent placement, is commonly used for the prevention and management of intestinal obstruction resulting from metastatic colonic stenosis [21]. Presently, the use of S-1 predominates in most cases, possibly due to its widespread use in Japan, where GC cases are more prevalent. Targeted therapies and immunotherapy for metastatic GC have received increased attention in recent years [43]. However, results of targeted and immunological tests were not mentioned due to early case inclusion and incomplete data. It is recommended to improve microsatellite and HER-2 indicators to guide further treatment.

### Survival

Larger-scale clinical studies reporting overall 5-year survival rates for Schnitzler’s metastasis are still lacking [32]; however, our study revealed a median survival time of 16 months (95% CI: 9-NA), following diagnosis of metastatic cancer. Rectal metastasis manifests with various symptoms, significantly affecting the physical and psychological well-being of patients. Therefore, we believe that the prognosis of patients with Schnitzler’s metastasis is characterized by decreased OS rates and reduced quality of life. Regular follow-up is crucial in managing patients with rectal metastases from GC, and it facilitates the early detection of tumor recurrence, metastasis, or any treatment-related complications. Research should also aim to identify biomarkers for early screening, diagnosis, and prognosis.

### Limitations

Our review relies upon case series or case reports, thus entailing a substantial risk for systematic biases. Furthermore, the small sample size is a limitation inherent to case reports.

## Conclusions

In conclusion, Schnitzler’s metastasis is uncommon, and its identification via colonoscopy pathology poses challenges, potentially leading to treatment delays. Imaging features, such as concentric thickening of the intestinal wall with notable layered enhancement, can aid in diagnosis. Nonetheless, deep puncture biopsy of bowel lesions remains the diagnostic gold standard for rectal metastases, particularly in patients with symptoms of mechanical bowel obstruction. Accurate differentiation between rectal metastasis and primary rectal cancer is imperative to prevent unnecessary therapeutic interventions.

## Statements and declarations

### Data availability statement

Available upon request to the corresponding author.
